# Sarcopenia risk assessment among physically inactive middle-aged and older adults: interpretable machine-learning models in UK and US cohorts

**DOI:** 10.1017/S1463423626101364

**Published:** 2026-06-24

**Authors:** Zhenhao Lin, Young-Je Sim, Chuang Zhang, Kunpeng Wu, Zhonghua Sun, Yuwen ShangGuan, Litao Yan

**Affiliations:** 1 Kunsan National University, Republic of Korea; 2 Hanyang University - Seoul Campus: Hanyang University, Republic of Korea; 3 Medical School of Nanjing University: Nanjing University Medical School, China; 4 Changzhou Maternal and Child Health Care Hospital: Changzhou Women and Children, China

**Keywords:** ELSA, machine learning, NHANES, physical inactivity, risk assessment, sarcopenia

## Abstract

**Aim:**

To develop and assess interpretable machine-learning models for sarcopenia risk assessment among physically inactive middle-aged and older adults using two large population-based datasets from the UK and the US.

**Background:**

Physical inactivity represents a major modifiable risk factor for sarcopenia in aging populations, yet prediction models specifically targeting this high-risk subgroup remain limited. This study developed and evaluated interpretable machine-learning models for sarcopenia risk stratification in physically inactive middle-aged and older adults using large-scale UK and US population-based data.

**Methods:**

We analyzed physically inactive participants from the English Longitudinal Study of Ageing (ELSA, 2012; *n* = 1,146) and the US National Health and Nutrition Examination Survey (NHANES, 1999–2006 and 2011–2018; *n* = 2,733). Sarcopenia and physical inactivity were defined using cohort-specific measurements and cutoffs. Within each cohort, six machine-learning algorithms were trained using 70/30 training–testing splits, Synthetic Minority Oversampling Technique to address class imbalance, and five-fold cross-validation for hyperparameter optimization. Model performance was evaluated using area under the curve, accuracy, precision, recall, and F1 scores. Shapley Additive Explanations quantified predictor contributions, and stratified analyses explored heterogeneity by age and body-composition strata.

**Findings:**

Random forest demonstrated optimal performance across both cohorts (area under the curve: 0.817 and 0.801; accuracy: 83.8% and 83.1%). Shapley Additive Explanations analysis revealed waist-to-height ratio as the dominant predictor, followed by age, frailty score, and poverty-income ratio. Stratified analyses showed heterogeneous risk patterns across age groups and body-composition categories.

## Graphical abstract

**Figure:**
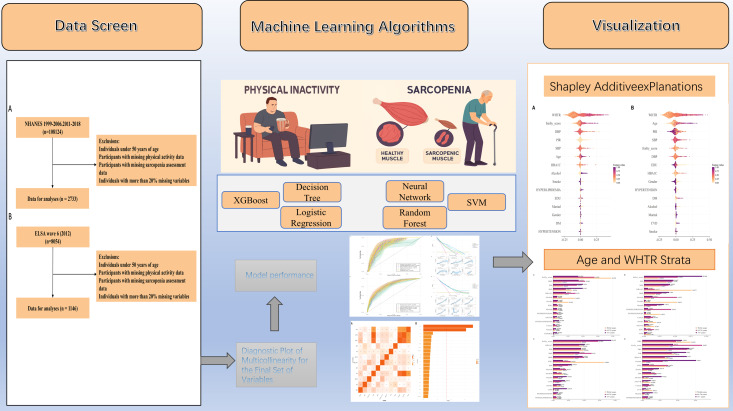

## Introduction

Sarcopenia is a geriatric syndrome characterized by progressive declines in skeletal muscle mass and strength and has been officially recognized in the ICD-10-CM, with a high prevalence among middle-aged and older adults (Hägglund *et al*., 2019). It increases risks of falls, hospitalization, and mortality and is closely linked with chronic conditions such as diabetes, cardiovascular disease, and cognitive impairment (Cruz-Jentoft *et al*., 2010; Kilaitė *et al.*, 2025), representing a growing healthcare challenge.

Physical inactivity is one of the most common and modifiable risk factors for sarcopenia and can accelerate muscle loss through metabolic, inflammatory, and endocrine pathways (Mo *et al*., 2024; Tsai *et al*., 2024). With the increasing adoption of sedentary lifestyles, the prevalence of physical inactivity continues to rise globally, especially among middle-aged and older adults, making this subgroup an emerging priority in muscle health management (Evans *et al*., 2024). However, evidence on sarcopenia risk assessment specifically targeting physically inactive individuals remains limited. More specifically, three gaps remain insufficiently addressed. First, existing sarcopenia risk-assessment models have mostly been developed in general aging populations, rather than in physically inactive adults as a distinct high-risk subgroup. Second, most available models have been derived from single datasets or single healthcare settings, leaving uncertainty about whether model performance and key risk patterns are consistent across different populations and diagnostic frameworks. Third, many machine-learning studies have focused mainly on discrimination metrics, with limited attention to interpretability and clinical translation. These limitations restrict the ability to identify high-risk individuals and to understand which modifiable factors may guide targeted prevention (Gao *et al*., 2021).

Cross-national differences in healthcare systems and lifestyle patterns may further influence recognition and prediction of sarcopenia (Beaudart *et al*., 2023). The UK and US share similarities as high-income countries but differ substantially in healthcare systems, socioeconomic inequality, and physical activity patterns (Sato *et al*., 2020; Lynch *et al*., 2024). Comparative risk-stratification research in these two settings can therefore improve understanding of whether key risk patterns are consistent across populations and inform targeted interventions.

Traditional statistical models often struggle with high-dimensional and nonlinear relationships (Coupland *et al*., 2025). Machine learning methods can address these limitations (Leghissa *et al*., 2024), but most sarcopenia-related ML studies rely on single models, lack cross-population evaluation across different healthcare settings, and remain insufficiently interpretable (Urzi *et al*., 2025). The Shapley Additive Explanations (SHAP) framework enhances transparency by quantifying feature contributions (Qi *et al*., 2025).

To address these gaps, we used NHANES and ELSA data to identify physically inactive adults aged 50 years and older, systematically compared major machine-learning algorithms for sarcopenia risk assessment, examined the consistency of model performance and key contributors across UK and US datasets, and applied SHAP to improve model interpretability. We also developed an online risk-assessment tool based on the selected model to facilitate individualized sarcopenia risk stratification in this high-risk population.

## Methods

### Data source and study population

**Figure 1.**
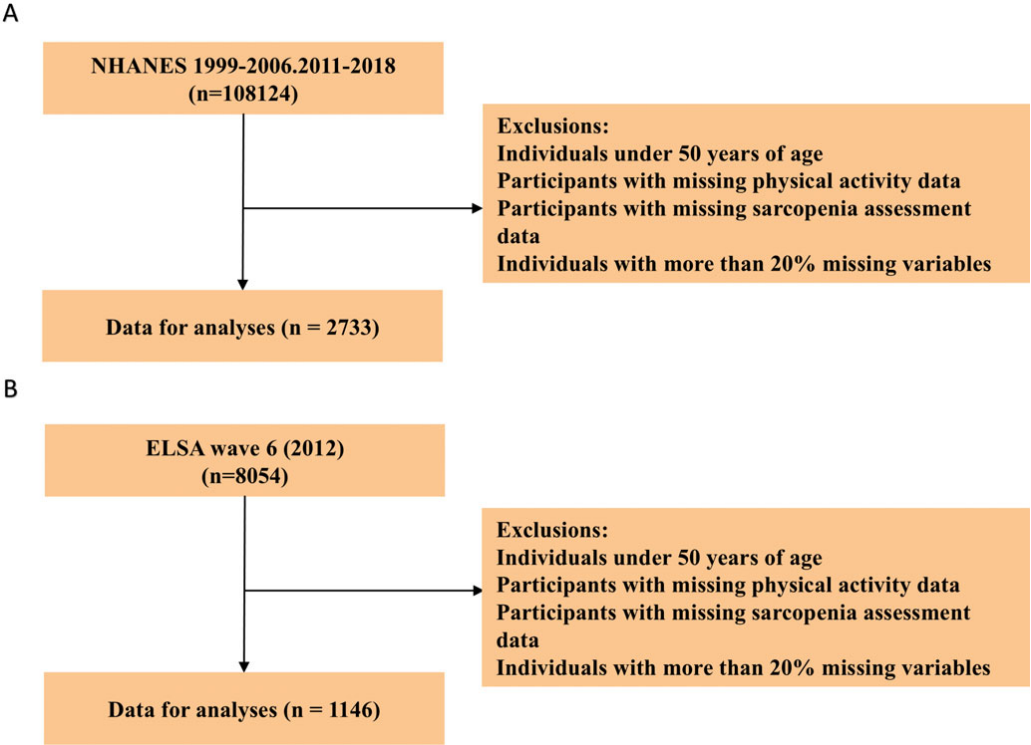
Population screening process.

This study was based on two major prospective cohort datasets: the National Health and Nutrition Examination Survey (NHANES) in the United States and the English Longitudinal Study of Aging (ELSA) in the United Kingdom, with the aim of systematically characterizing the health profiles of physically inactive middle-aged and older adults. A total of 108,124 respondents from NHANES (1999–2006 and 2011–2018 cycles) and 8,054 respondents from the sixth wave of ELSA, conducted in 2012, were initially included. Participants younger than 50 years, pregnant women, and individuals with missing key variables were subsequently excluded using harmonized criteria, resulting in final analytical samples of 2,733 physically inactive individuals in NHANES and 1,146 in ELSA. Both cohorts provided multidimensional information, including sociodemographic characteristics, clinical health indicators, nutritional measures, and physical activity variables, and all data collection procedures were approved by relevant ethics committees, with written informed consent obtained from all participants. The detailed participant selection flow is illustrated in Figure 1.

### Definition of physical inactivity

Physical activity (PA) was assessed using self-reported questionnaires in both cohorts. In NHANES, participants reported work-related, transportation, and recreational activities, including frequency and duration (Wanner *et al*., 2017; Wang and Deng, 2025). PA intensity was quantified as weekly MET-minutes (MET × duration × frequency) (Jetté *et al.*, 1990), and insufficient PA was defined as <600 MET-minutes/week according to WHO guidelines (Wanner *et al.*, 2017). Participants with missing PA data or MET totals ≥600 were excluded. In ELSA, detailed information required to calculate MET-minutes was unavailable. Instead, PA was assessed by the frequency of moderate or vigorous activity, and individuals reporting ‘hardly ever or never’ were classified as physically inactive (Yan *et al*., 2026).

### Definition of sarcopenia

Given the differences in clinical guidelines between the UK and US, region-specific diagnostic criteria were strictly applied to ensure the clinical relevance of the model within each healthcare system. In NHANES, appendicular lean mass (ALM) measured by DXA was used, and sarcopenia was defined using the ALM/BMI ratio based on FNIH cutoffs (<0.789 kg/m^2^ for men and <0.512 kg/m^2^ for women) (Studenski *et al*., 2014). In ELSA, sarcopenia followed the revised EWGSOP2 definition, requiring both low muscle strength and low skeletal muscle mass (Pavón-Pulido *et al*., 2024). Low strength was defined as handgrip <27 kg in men and <16 kg in women. Skeletal muscle mass was estimated using the Lee equation and standardized to BMI to derive skeletal muscle index (SMI), with low SMI defined as the lowest sex-specific quartile.

### Machine learning data preprocessing

The initial dataset in this study consisted of 24 features, including 11 continuous and 13 categorical variables, covering multiple dimensions such as sociodemographic characteristics, clinical health status, nutritional structure, and physical activity levels. All preprocessing procedures were performed separately within the ELSA and NHANES cohorts. Variables with more than 20% missingness were excluded within each cohort before model development. For the remaining variables, each cohort was randomly divided into training and test sets using a 70:30 split. To avoid information leakage, missing-value imputation was performed after the training–test split. Specifically, a random forest–based imputation procedure implemented using the ‘missForest’ package in R was fitted using the training data only, with 100 trees and a maximum of 10 iterations. The fitted imputation procedure was then applied to the corresponding test set. Imputation accuracy was evaluated in the training data using the normalized root mean squared error (NRMSE) for continuous variables and the proportion of falsely classified entries (PFC) for categorical variables. To reduce multicollinearity, variable-selection procedures were also conducted within the training data only and separately within each cohort. Pearson correlation coefficients were first calculated only among continuous variables, because this analysis was intended to identify strong linear correlations between continuous features. Variables with strong correlations were identified using an absolute correlation threshold of |*r*| > 0.85. This threshold was selected to detect highly redundant variables while avoiding unnecessary exclusion of moderately correlated features with potential clinical relevance. When multiple anthropometric indicators were highly correlated, WHTR was retained because it was clinically interpretable, available in both cohorts, and directly relevant to central adiposity and sarcopenia risk assessment; waist circumference, BRI, and WWI were therefore excluded from the final modeling set. Variance inflation factors (VIFs) were then calculated for the remaining candidate variables in the training set, with categorical variables dummy-coded where appropriate. A VIF threshold of >5 was used to identify potentially problematic multicollinearity and improve model stability. When conceptually overlapping variables showed elevated VIF values, the variable with broader clinical interpretability and cross-cohort availability was retained.

### Development and evaluation of predictive models

Within each cohort, the dataset was randomly split into training and test sets at a ratio of 70:30, and class imbalance was addressed by applying the Synthetic Minority Oversampling Technique (SMOTE) only to the training set (Supplementary Figure 1). The six algorithms were selected to represent commonly used modeling families with different assumptions and levels of complexity. Logistic Regression was included as a conventional linear baseline model, Decision Tree as an interpretable tree-based model, Random Forest and XGBoost as ensemble tree-based methods capable of capturing nonlinear relationships and interactions, SVM as a kernel-based classifier, and Neural Network as a flexible nonlinear modeling approach. This selection allowed comparison between parsimonious conventional approaches and more complex machine-learning methods. The six models were trained separately in each cohort. Hyperparameters were optimized using grid search with five-fold cross-validation in the training set, with mean cross-validated AUC used as the optimization criterion. Model evaluation was performed on the independent test set and included AUC with 95% confidence intervals, accuracy, precision, recall, and F1 score. Model performance was visualized using ROC curves, decision curve analysis (DCA), and calibration plots. To improve interpretability and identify major contributors to model-estimated sarcopenia risk, SHAP analyses were applied to the selected best-performing model using the shapviz package, with further stratified comparisons of key SHAP-derived variables across cohorts. Finally, an interactive risk-assessment tool was developed using R Shiny to enable individualized sarcopenia risk estimation among physically inactive middle-aged and older adults.

### Statistical methods

Statistical analyses were conducted using R software (version 4.5.1). The normality of continuous variables was assessed using the Shapiro–Wilk test. Variables following a normal distribution were summarized as mean ± standard deviation, and between-group differences were examined using independent-samples *t* tests. Variables not conforming to normality were presented as median with interquartile range, and group comparisons were performed using the Mann–Whitney U test. Categorical variables were expressed as counts and percentages, and differences between groups were assessed using the chi-square test. A two-sided *p* value < 0.05 was considered statistically significant for all baseline comparisons. Model evaluation was performed on the independent test set. Discriminative performance was assessed using the area under the receiver operating characteristic curve (AUC), with 95% confidence intervals estimated using the DeLong method. Accuracy, precision, recall, and F1 score were also calculated to evaluate classification performance. Calibration was assessed using calibration plots and Brier scores, with lower Brier scores indicating better agreement between predicted probabilities and observed outcomes. DCA was performed to evaluate the clinical utility of each model by estimating net benefit across a range of threshold probabilities and comparing model-based strategies with treat-all and treat-none strategies.

## Results

### Baseline characteristics

This study included 1,146 physically inactive participants from the ELSA cohort and 2,733 participants from the NHANES cohort. In the NHANES cohort, individuals with sarcopenia were older and predominantly male, whereas no significant sex differences were observed in the ELSA cohort. Both cohorts showed that participants with sarcopenia had lower poverty-income ratios. In both datasets, sarcopenia was associated with higher prevalence of diabetes and hypertension, and the NHANES data further indicated associations with cardiovascular disease, stroke, and atherosclerosis. Regarding body composition, individuals with sarcopenia exhibited higher waist circumference, waist-to-height ratio (WHTR), body roundness index (BRI), a body shape index (ABSI), and lower weight-adjusted waist index (WWI) in NHANES cohort, along with elevated frailty scores in both cohorts. In the NHANES cohort, individuals with sarcopenia also exhibited higher systolic and diastolic blood pressure, as well as elevated glycated hemoglobin levels. Detailed data for both groups are presented in Supplementary Table 1 and Supplementary Table 2.

### Machine learning model construction and comparison

**Figure 2.**
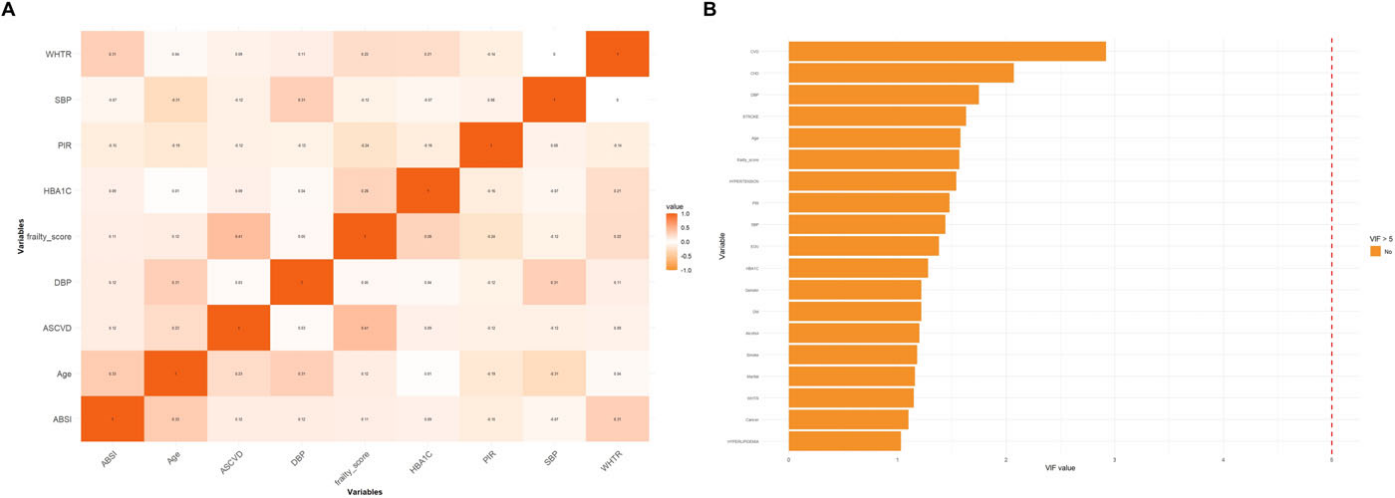
Diagnosis of multicollinearity in the final variable set. (A) Heatmap of Pearson correlation matrix; (B) Variance inflation factor plot.

Before model construction, Pearson correlation coefficients were calculated among continuous variables to reduce multicollinearity (Supplementary Figure 2). The Pearson correlation analysis revealed substantial multicollinearity among anthropometric variables, with an almost perfect correlation observed between WHTR and waist circumference (*r* = 0.903), as well as between WHTR and BRI (*r* = 0.995). In addition, the WWI showed strong correlations with waist circumference (*r* = −0.857) and WHTR (*r* = −0.991). To reduce redundancy and avoid instability caused by multicollinearity, WAIST, BRI, and WWI were removed from subsequent modeling. VIFs were then computed for the remaining variables to assess residual multicollinearity (Supplementary Figure 3). The results showed that CVD (VIF = 9.25) and ASCVD (VIF = 10.74) exceeded the commonly accepted threshold of 5, indicating severe multicollinearity, and therefore ASCVD was excluded from the final model. After addressing multicollinearity, a total of 20 variables – including 13 categorical and 8 continuous features – were retained for modeling (Figure 2), ensuring adequate model stability and interpretability.

Six machine-learning models were developed separately in the ELSA and NHANES cohorts for sarcopenia risk assessment among physically inactive middle-aged and older adults (Table 1). In the ELSA cohort, logistic regression, XGBoost, and random forest demonstrated comparable and superior discriminative performance, with AUCs of 0.818, 0.818, and 0.817, respectively, while random forest achieved the best balance across performance metrics (accuracy 0.838, precision 0.909, recall 0.903, and F1 score 0.906). In the NHANES cohort, random forest again exhibited the highest performance (AUC = 0.801), with an accuracy of 0.831, precision of 0.874, recall of 0.923, and an F1 score of 0.898. Decision trees performed the weakest in both cohorts (AUC = 0.707 in ELSA and 0.726 in NHANES). Despite differences in PA definitions and diagnostic criteria for sarcopenia across the two cohorts, both demonstrated that ensemble learning methods such as random forest and XGBoost consistently yielded superior discriminative ability, suggesting relatively consistent model performance across heterogeneous populations.

**Table 1.** Machine learning model performance evaluation in ELSA and NHANES cohorts Table 1 long description.

Model	Cohort	AUC (95% CI)	Accuracy	Precision	Recall	F1 Score
Random forest	ELSA	0.817 (0.762–0.872)	0.838	0.909	0.903	0.906
	NHANES	0.801 (0.764–0.839)	0.831	0.874	0.923	0.898
XGBoost	ELSA	0.818 (0.764–0.872)	0.829	0.908	0.893	0.900
	NHANES	0.794 (0.755–0.833)	0.811	0.882	0.884	0.883
Logistic regression	ELSA	0.818 (0.761–0.876)	0.817	0.930	0.852	0.890
	NHANES	0.792 (0.754–0.829)	0.787	0.878	0.855	0.866
SVM	ELSA	0.800 (0.739–0.860)	0.864	0.864	1.000	0.927
	NHANES	0.786 (0.747–0.824)	0.800	0.884	0.866	0.875
Neural network	ELSA	0.809 (0.753–0.866)	0.757	0.950	0.758	0.843
	NHANES	0.782 (0.745–0.819)	0.705	0.907	0.707	0.795
Decision tree	ELSA	0.707 (0.621–0.793)	0.780	0.902	0.836	0.868
	NHANES	0.726 (0.685–0.768)	0.805	0.853	0.917	0.884

**Figure 3.**
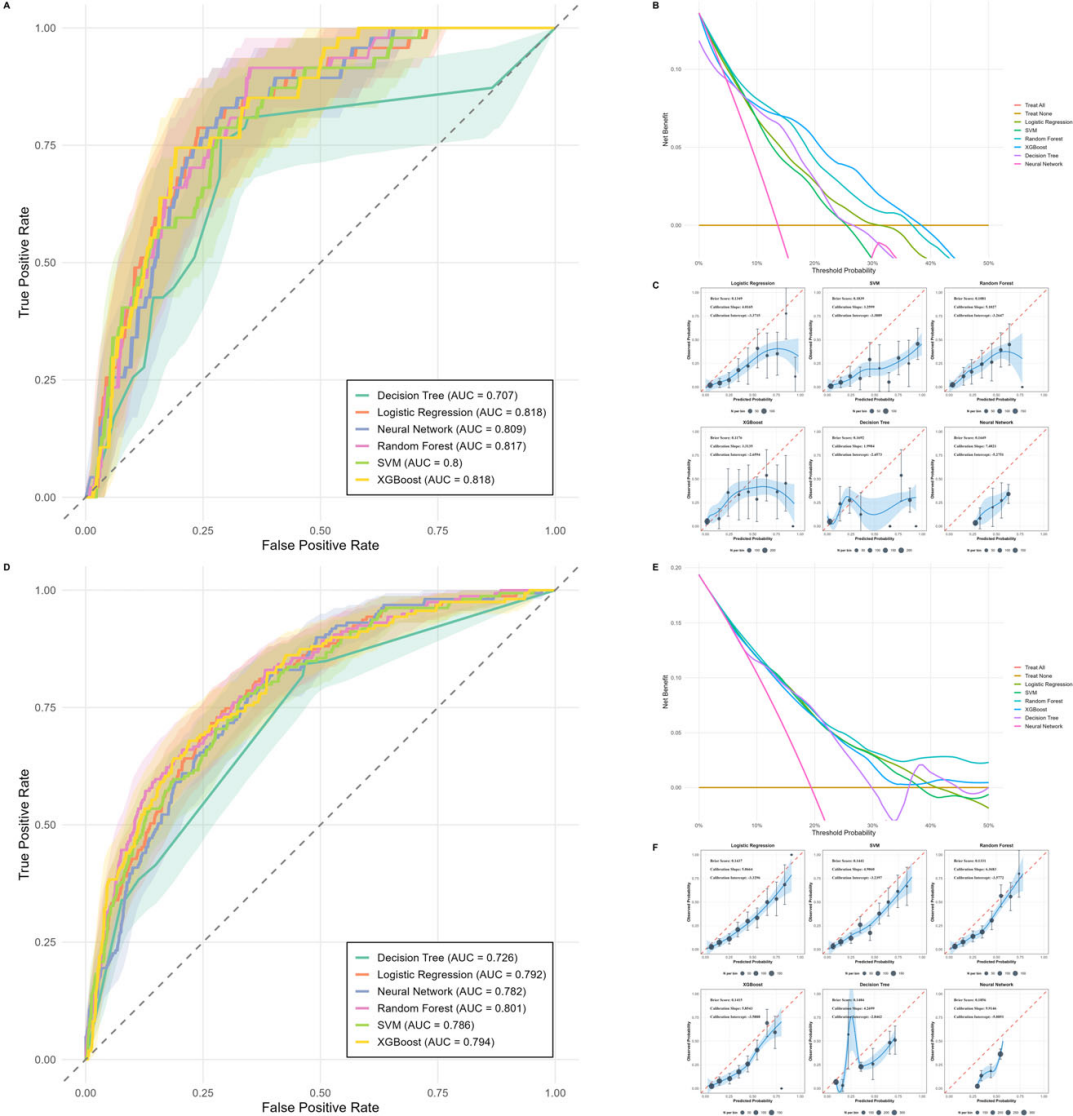
Performance comparison of six machine learning models in ELSA (A–C) and NHANES (D–F) cohorts. (A, D) ROC curves; (B, E) Decision curve analysis; (C, F) Calibration curves.

Figure 3 presents a comprehensive comparison of the six models across both cohorts. The ROC curves (Figure 3A, 3D) show that random forest achieved the highest AUC in both ELSA (0.817) and NHANES (0.801). Decision curve analysis (Figure 3B, 3E) indicates that random forest provided the greatest net benefit within the 20–30% threshold range, avoiding approximately 45 and 50 unnecessary interventions per 1,000 individuals at a 20% threshold in the ELSA and NHANES cohorts, respectively. Calibration curves (Figure 3C, 3F) further confirmed the favorable calibration performance of random forest, with the lowest Brier scores in both cohorts (0.119 and 0.138), indicating excellent agreement between model-estimated probabilities and observed outcomes. Considering discriminative performance, overall metrics, clinical net benefit, and calibration, random forest was identified as the best-performing model for predicting sarcopenia risk among physically inactive older adults in both cohorts. Training-set performance and optimized hyperparameters are provided in Supplementary Tables 3 and 4.

### Importance of SHAP values in interpreting the best models across ELSA and NHANES

**Figure 4.**
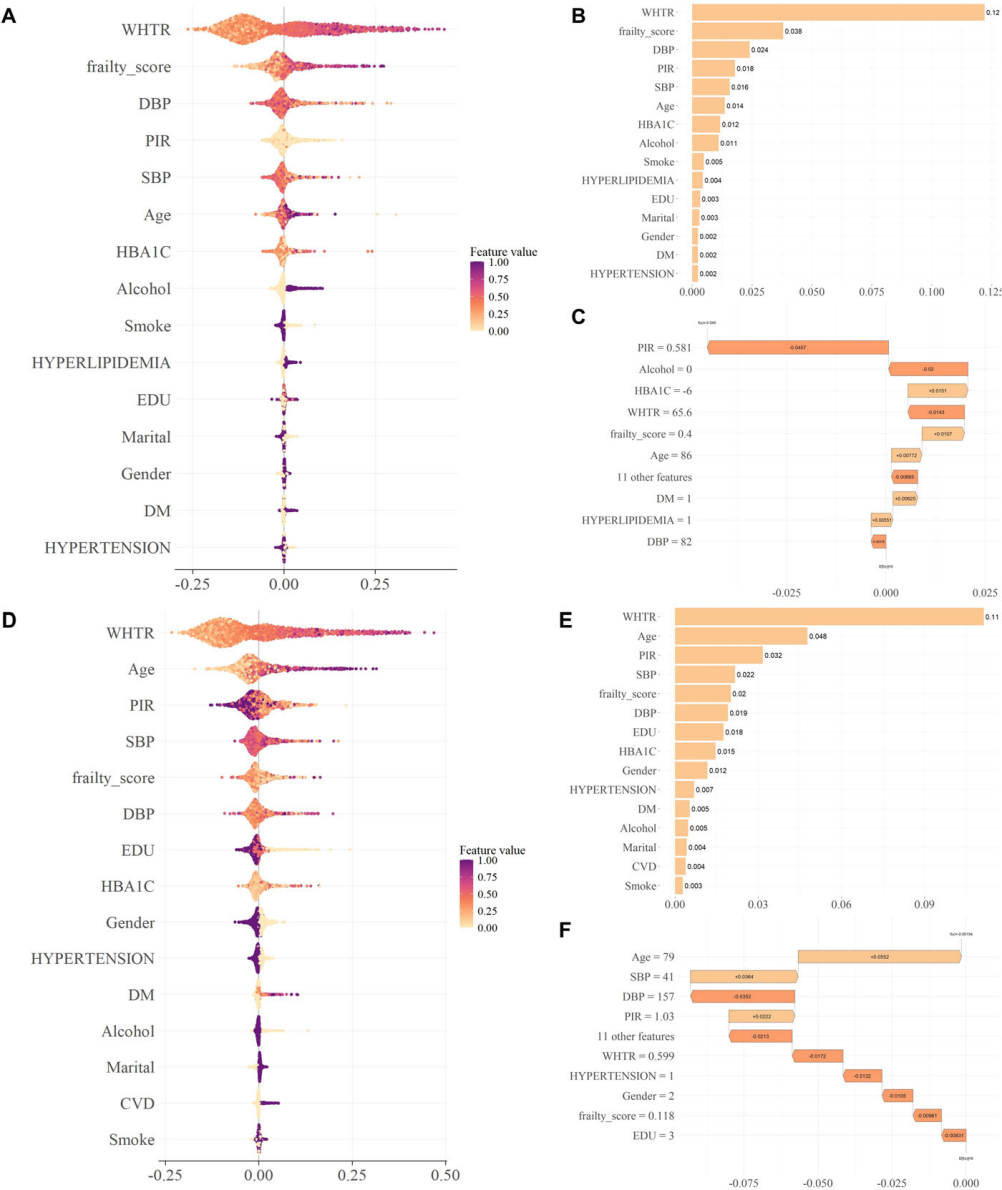
Figure 4 long description. (A) SHAP summary in ELSA; (B) Feature importance in ELSA; (C) SHAP waterfall in ELSA; (D) SHAP summary in NHANES; (E) Feature importance in NHANES; (F) SHAP waterfall in NHANES.

To gain deeper insights into the prediction mechanisms of the best-performing models in both cohorts, SHAP analyses were conducted and integrated into a single figure. As shown in Figure 4, the SHAP summary and feature importance plots demonstrated that WHTR was the most influential contributor to model-estimated sarcopenia risk in both ELSA and NHANES, with mean absolute SHAP values of approximately 0.12 and 0.11, respectively, substantially exceeding those of other features. In the ELSA cohort, frailty score, DBP, and PIR followed in descending order of importance, whereas in the NHANES cohort, age, PIR, SBP, and frailty score constituted the subsequent top-ranked predictors.

The waterfall plots (Figure 4C and 4F) further illustrated how individual predictors cumulatively influenced model output at the individual level. In both cohorts, higher age, elevated WHTR values, and greater frailty levels contributed positively to sarcopenia risk, whereas higher PIR values generally exerted negative contributions. Collectively, these findings highlight the central role of central adiposity and sociodemographic factors (e.g., PIR and age) in shaping sarcopenia risk among physically inactive older adults. The SHAP dependence plots for the top six predictors in each cohort are provided in Supplementary Figures S4 and S5 for additional visualization of nonlinear relationships and interaction patterns.

### Heterogeneity of model-predicted risk across age and WHTR strata

**Figure 5.**
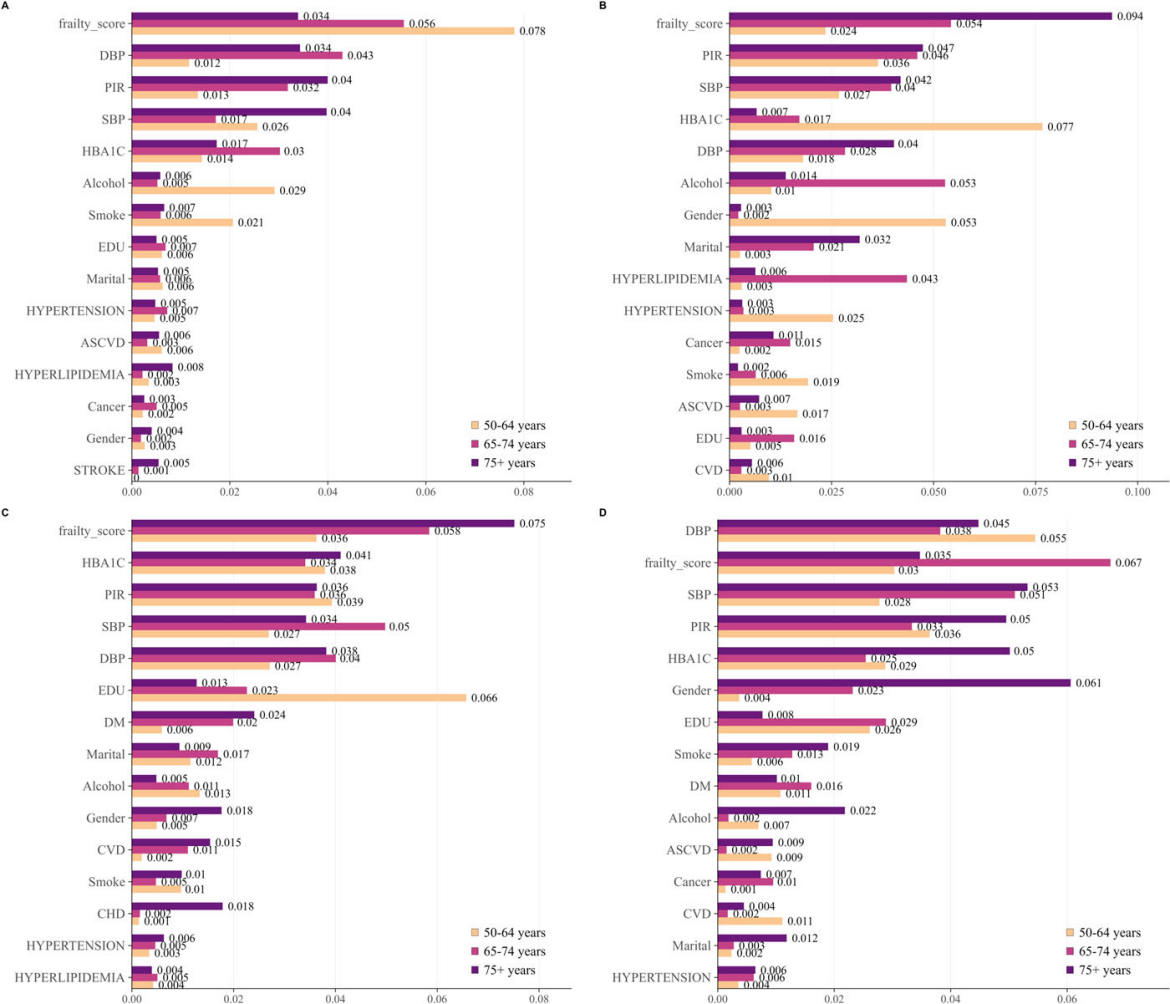
Heterogeneity of model-predicted risk across age and WHTR strata in ELSA and NHANES. (A) Age-stratified analysis among ELSA participants without central obesity (normal WHTR, <0.50). (B) Age-stratified analysis among ELSA participants with central obesity (WHTR ≥ 0.50). (C) Age-stratified analysis among NHANES participants without central obesity (normal WHTR, <0.50). (D) Age-stratified analysis among NHANES participants with central obesity (WHTR ≥ 0.50).

To explore how predictive factors behaved across subgroups, stratified analyses were conducted by age (50–64, 65–74, ≥75 years) and WHTR (<0.50 vs ≥0.50) (Figure 5). Compared with the overall population results where WHTR dominated, distinct predictive patterns emerged within each stratum. In the ELSA cohort, among individuals with normal WHTR (Figure 5A), frailty score was the strongest predictor across all age groups, with the highest contribution observed in the 50–64 age group (0.078). PIR became more influential in the ≥75 age group (0.040), whereas DBP was most prominent in those aged 65–74 years (0.043). Among individuals with central obesity (Figure 5B), frailty score peaked in importance among those ≥75 years (0.094), while SBP played a notable role in older obese participants (0.042). In the NHANES cohort, the normal WHTR group (Figure 5C) similarly showed frailty score as the primary predictor, with the greatest contribution in the ≥75 age group (0.075). HBA1C showed a substantial contribution among individuals ≥75 years (0.041), while education level was notably important in the 50–64 age group (0.066). In the central obesity group (Figure 5D), DBP was most prominent among those aged 65–74 years (0.055), frailty score remained highly influential in both the 65–74 and ≥75 age groups, and HBA1C maintained substantial importance in older obese individuals (0.050). Comparison across cohorts revealed consistent age-dependent patterns for frailty score, PIR, and blood pressure measures among individuals with normal WHTR, whereas metabolic indicators played a more dominant role in the NHANES cohort among those with central obesity.

### Development and clinical application of online predictive tools

Building on the best-performing models developed earlier, we created a web-based risk-assessment tool (https://zhenhaolin.shinyapps.io/ELSA_NHANES_APP/) that enables dynamic estimation of an individual’s probability of sarcopenia. The tool allows users to select either the ELSA or NHANES dataset and performs individualized risk assessment based on the corresponding optimized random forest model and its key predictors. After selecting the appropriate dataset according to the target population and entering individual health indicators, the system computes the model-estimated probability of sarcopenia in real time and visualizes the contribution of each predictor through SHAP values.

## Discussion

By developing and comparing interpretable machine-learning models, this study addresses a critical gap in sarcopenia risk assessment specifically for physically inactive middle-aged and older adults. Using two large international datasets, we systematically compared six machine-learning algorithms for sarcopenia risk assessment among physically inactive middle-aged and older adults. Random Forest showed favorable overall performance in both the U.K. and U.S. cohorts, with AUC values of 0.817 and 0.801, respectively. However, its advantage over simpler models was modest, particularly in the ELSA cohort, where Logistic Regression and XGBoost achieved nearly identical AUC values. Therefore, Random Forest should not be interpreted as universally superior to traditional regression approaches. Rather, it was selected as the final model because it provided a favorable balance across discrimination, classification metrics, calibration, decision-curve performance, and cross-cohort consistency. These findings suggest that Random Forest may be useful for flexible risk stratification, whereas simpler models such as Logistic Regression may remain preferable when parsimony, transparency, ease of implementation, and direct coefficient interpretation are prioritized. Thus, the choice between traditional regression and machine-learning approaches should be guided by the intended clinical or public-health application rather than by discrimination metrics alone.

SHAP-based interpretability analyses identified WHTR, age, frailty score, and PIR as the most influential contributors to model-estimated sarcopenia risk. Importantly, these SHAP-derived variables should be interpreted as contributors to model output rather than as independent causal determinants. Some variables may also capture overlapping biological or clinical constructs. For example, WHTR, waist circumference, BRI, and WWI reflect related aspects of body size and central adiposity, whereas frailty score may partially overlap with functional decline and muscle weakness, which are closely related to sarcopenia itself. Therefore, the interpretation of these variables should consider both their biological plausibility and their potential conceptual overlap with sarcopenia-related phenotypes. Stratified analyses revealed heterogeneous risk patterns across age groups and WHTR categories, highlighting the importance of individualized risk assessment. Together, these findings provide a framework for sarcopenia risk stratification and targeted screening in physically inactive middle-aged and older adults.

WHTR, as an indicator of central obesity, demonstrated the strongest predictive capability in both cohorts. Previous studies have shown that visceral fat accumulation not only physically displaces muscle tissue but also accelerates muscle protein catabolism and loss through the release of pro-inflammatory cytokines (e.g., IL-6, TNF-α) and adipokine dysregulation, thereby contributing to a vicious cycle of ‘sarcopenic obesity’ (Bian *et al*., 2017). Comparison of SHAP dependence plots revealed a strong positive association between WHTR and predicted risk in both cohorts, although the magnitude of this effect was more pronounced in the NHANES cohort. This difference may reflect the higher prevalence of obesity and greater visceral fat accumulation in the U.S. population. Epidemiological data indicate that approximately 42% of U.S. adults are obese, compared with about 28% in the U.K., and the proportion of central obesity is also substantially higher in the U.S. population (Emmerich *et al*., 2024; Stiebahl, 2025), which may explain why WHTR serves as a more sensitive predictor in the NHANES cohort. WHTR may be particularly informative because it captures central adiposity rather than general body size alone. Although BMI is widely used, it does not distinguish fat mass from lean mass or reflect fat distribution, which is especially problematic in middle-aged and older adults experiencing age-related muscle loss and body-composition changes. In contrast, WHTR adjusts waist circumference for height and may better represent visceral fat accumulation, which is closely related to inflammation, insulin resistance, and impaired muscle metabolism. Therefore, the consistent importance of WHTR in both cohorts suggests that central adiposity may be more relevant than overall adiposity for identifying sarcopenia risk among physically inactive adults (Ashwell *et al*., 2012; Ashwell and Gibson, 2014).

Age made a substantial contribution to model-estimated sarcopenia risk in both cohorts, with muscle mass declining at an approximate rate of 1–2% per year with advancing age, a process regulated by multiple biological mechanisms, including motor neuron loss, reduced satellite cell regenerative capacity, mitochondrial dysfunction, elevated chronic inflammation, and diminished secretion of anabolic hormones such as testosterone and growth hormone (Keller and Engelhardt, 2014). Among physically inactive middle-aged and older adults, age-related muscle decline may act synergistically with insufficient mechanical stimulation.

Frailty score also ranked highly among the predictive variables in both cohorts, as frailty and sarcopenia share substantial overlap, with both conditions involving similar pathophysiological mechanisms, including chronic inflammation, mitochondrial dysfunction, increased oxidative stress, and imbalances between protein synthesis and degradation (Rodríguez-Mañas *et al*., 2021). Frailty score typically integrates multidimensional information – such as gait speed, grip strength, physical activity, fatigue, and unintentional weight loss – providing a comprehensive reflection of an individual’s physiological reserve and degree of functional decline. Among physically inactive individuals, the vicious cycle between disuse-related muscle atrophy and frailty is even more pronounced, as reduced physical activity leads to declines in muscle mass and function, which in turn further limits mobility and accelerates the progression of frailty.

Socioeconomic indicators, including education level and PIR, also demonstrated meaningful predictive value in the models, which aligns with findings from the broader health inequalities literature. Lower socioeconomic status is often associated with multiple adverse conditions, such as inadequate nutritional intake, limited access to healthcare, low health literacy, and elevated chronic stress levels (Lago *et al*., 2018). In the NHANES cohort, PIR had the third-highest SHAP value (0.03), reflecting the substantial impact of income inequality on health outcomes in the U.S. context. Individuals with lower incomes may face food insecurity, insufficient intake of high-quality protein, and limited access to exercise facilities, all of which represent potential risk factors for sarcopenia (Gandham *et al*., 2021). Education level is closely linked to health literacy, and individuals with lower educational attainment may lack knowledge related to nutrition, physical activity, and disease prevention, making it more difficult for them to adopt and sustain healthy lifestyle behaviors.

Chronic disease indicators, such as diabetes, hypertension, hyperlipidemia, and cardiovascular disease, exhibited varying levels of predictive value in the models. The association between diabetes and sarcopenia has been well established, with hyperglycemia accelerating muscle protein degradation through the accumulation of advanced glycation end-products (AGEs), insulin resistance, and heightened inflammatory responses (Feng *et al*., 2022). Hypertension and cardiovascular disease may indirectly impair muscle function by affecting muscle perfusion and oxygen delivery. Moreover, chronic diseases and sarcopenia may share upstream risk factors – including obesity, inflammation, and sedentary behavior – which may attenuate their independent contributions after adjustment for other covariates (Zou *et al*., 2025).

The heterogeneity analysis provides clinically relevant information for individualized risk stratification. As shown in Figure 5, the relative importance of key contributors differed across age and WHTR strata, suggesting that a uniform screening strategy may not be appropriate for all physically inactive adults. Among individuals with normal WHTR, frailty score remained a leading contributor, indicating that functional assessment should be emphasized even when central obesity is absent. In contrast, among individuals with central obesity, cardiometabolic markers such as blood pressure and HbA1c showed greater importance in several age subgroups, suggesting that muscle health assessment should be integrated with metabolic risk evaluation in this population. The importance of PIR and education level further indicates that socioeconomic disadvantage may help identify individuals who could benefit from nutritional support, health education, and improved access to preventive care. These findings suggest that age-, body-composition-, functional-, metabolic-, and socioeconomic-informed risk stratification may support more targeted screening and individualized intervention strategies for physically inactive middle-aged and older adults.

Finally, based on our findings, we developed an online risk assessment tool that enables dynamic estimation of an individual’s likelihood of having sarcopenia. The tool allows users to select either the ELSA or NHANES dataset and performs personalized risk assessment using the corresponding optimized random forest model and key predictors. After choosing the appropriate dataset based on the characteristics of the target population and entering individual health metrics, the system computes the probability of sarcopenia in real time and visualizes the contribution of each predictor through SHAP-based interpretability plots.

However, this study has several limitations. First, the cross-sectional design allows only the identification of associations rather than causal relationships, and prospective cohort studies are needed to confirm these findings. Second, the two cohorts employed different diagnostic criteria for sarcopenia, with the FNIH definition used in NHANES and the EWGSOP2-based definition used in ELSA. This heterogeneity may have influenced sarcopenia prevalence, case classification, model performance, and the relative importance of predictors. Specifically, the FNIH definition emphasizes low appendicular lean mass adjusted for BMI, whereas the EWGSOP2-based definition incorporates low muscle strength and estimated skeletal muscle mass, thereby capturing partly different sarcopenia phenotypes. Accordingly, the observed consistency of major contributors, such as WHTR, frailty score, age, and socioeconomic indicators, should be interpreted as cross-cohort consistency in risk patterns rather than direct equivalence of absolute risk estimates. Further studies using harmonized diagnostic criteria and prospective external validation are needed to confirm the generalizability and clinical transportability of these models. Importantly, the fact that WHTR emerged as the dominant predictor under both definitions strongly attests to the biological robustness of our findings, suggesting that central obesity remains a universal risk factor regardless of the specific diagnostic framework used. But it also enhances the applicability of the model across varying diagnostic frameworks. Future research directions include conducting 5–10-year prospective follow-up studies to explore temporal relationships among predictors; performing external validation across diverse racial groups and healthcare systems to assess generalizability; evaluating the clinical utility of the prediction tool through randomized controlled trials; and applying multi-omics approaches to elucidate the molecular mechanisms underlying sarcopenia development.

## Conclusion

By analyzing data from the large ELSA and NHANES cohorts, this study focused on physically inactive middle-aged and older adults and developed interpretable machine-learning models for sarcopenia risk assessment and stratification. The models identified WHTR, age, PIR, and frailty score as important contributors to model-estimated sarcopenia risk, and stratified analyses further revealed heterogeneous risk patterns across age groups and body-composition categories. Additionally, a web-based risk-assessment tool was constructed to support visualization and individualized estimation of sarcopenia-related risk patterns in this high-risk population. Further prospective studies using harmonized diagnostic criteria and external clinical validation are needed before this tool can be recommended for routine screening or individualized intervention.

## Supporting information

10.1017/S1463423626101364.sm001 Lin et al. supplementary material 1 Lin et al. supplementary material

10.1017/S1463423626101364.sm002 Lin et al. supplementary material 2 Lin et al. supplementary material

10.1017/S1463423626101364.sm003 Lin et al. supplementary material 3 Lin et al. supplementary material

10.1017/S1463423626101364.sm004 Lin et al. supplementary material 4 Lin et al. supplementary material

10.1017/S1463423626101364.sm005 Lin et al. supplementary material 5 Lin et al. supplementary material

10.1017/S1463423626101364.sm006 Lin et al. supplementary material 6 Lin et al. supplementary material

10.1017/S1463423626101364.sm007 Lin et al. supplementary material 7 Lin et al. supplementary material

10.1017/S1463423626101364.sm008 Lin et al. supplementary material 8 Lin et al. supplementary material

10.1017/S1463423626101364.sm009 Lin et al. supplementary material 9 Lin et al. supplementary material

## Data Availability

All data used in this study are publicly available. ELSA data can be accessed via the UK Data Service (https://www.elsa-project.ac.uk/accessing-elsa-data), and NHANES data can be accessed via the CDC website (https://www.cdc.gov/nchs/nhanes/).

## Supplementary material

The supplementary material for this article can be found at https://doi.org/10.1017/S1463423626101364.

## Table 1. Long description

The table compares the performance of six machine-learning models in two cohorts, ELSA and NHANES, for sarcopenia risk assessment. It has 7 rows and 7 columns. The columns are labeled Model, Cohort, AUC (95% CI), Accuracy, Precision, Recall, and F1 Score. The rows are labeled with the names of the models: Random forest, XGBoost, Logistic regression, SVM, Neural network, and Decision tree. Each model's performance is evaluated in both the ELSA and NHANES cohorts. The values in the table include AUC with 95% confidence intervals, accuracy, precision, recall, and F1 score for each model and cohort. For example, the Random forest model in the ELSA cohort has an AUC of 0.817 (0.762–0.872), accuracy of 0.838, precision of 0.909, recall of 0.903, and F1 score of 0.906. In the NHANES cohort, the same model has an AUC of 0.801 (0.764–0.839), accuracy of 0.831, precision of 0.874, recall of 0.923, and F1 score of 0.898.

Navigate back to Table 1..

## Figure 4. Long description

Panel A: A SHAP summary plot for the ELSA cohort. The x-axis represents SHAP values ranging from -0.25 to 0.25, and the y-axis lists features such as WHTR, frailty_score, DBP, PIR, SBP, Age, HBA1C, Alcohol, Smoke, HYPERLIPIDEMIA, EDU, Marital, Gender, DM, and HYPERTENSION. The plot shows the distribution of SHAP values for each feature, with colors indicating feature values. Panel B: A bar graph showing feature importance in the ELSA cohort. The x-axis represents importance scores ranging from 0.000 to 0.120, and the y-axis lists the same features as in Panel A. WHTR has the highest importance score. Panel C: A SHAP waterfall plot for the ELSA cohort. The x-axis represents SHAP values ranging from -0.025 to 0.125, and the plot shows the contribution of each feature to the prediction for a specific instance, with PIR having the highest contribution. Panel D: A SHAP summary plot for the NHANES cohort. The x-axis represents SHAP values ranging from -0.25 to 0.50, and the y-axis lists features such as WHTR, Age, PIR, SBP, frailty_score, DBP, EDU, HBA1C, Gender, HYPERTENSION, DM, Alcohol, Marital, CVD, and Smoke. The plot shows the distribution of SHAP values for each feature, with colors indicating feature values. Panel E: A bar graph showing feature importance in the NHANES cohort. The x-axis represents importance scores ranging from 0.000 to 0.110, and the y-axis lists the same features as in Panel D. WHTR has the highest importance score. Panel F: A SHAP waterfall plot for the NHANES cohort. The x-axis represents SHAP values ranging from -0.075 to 0.025, and the plot shows the contribution of each feature to the prediction for a specific instance, with Age having the highest contribution.

Navigate back to Figure 4..
