# Supplementary figures and images for: Sarcopenia risk assessment among physically inactive middle-aged and older adults: interpretable machine-learning models in UK and US cohorts
Source: Prim Health Care Res Dev. 2026 Jun 24;27:e71. doi: 10.1017/S1463423626101364 (PMC13319488; doi:10.1017/S1463423626101364)

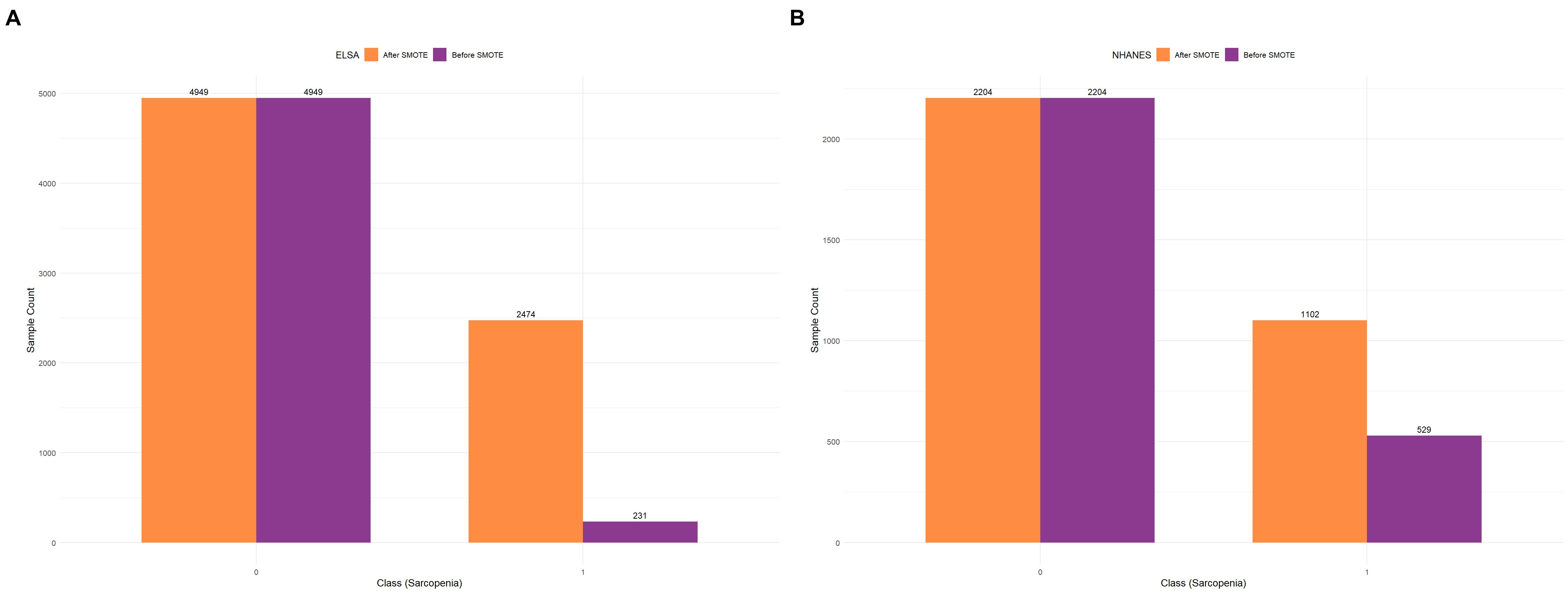

Supplement: Lin et al. supplementary material 1 — Lin et al. supplementary material [file S1463423626101364sup001.tiff]

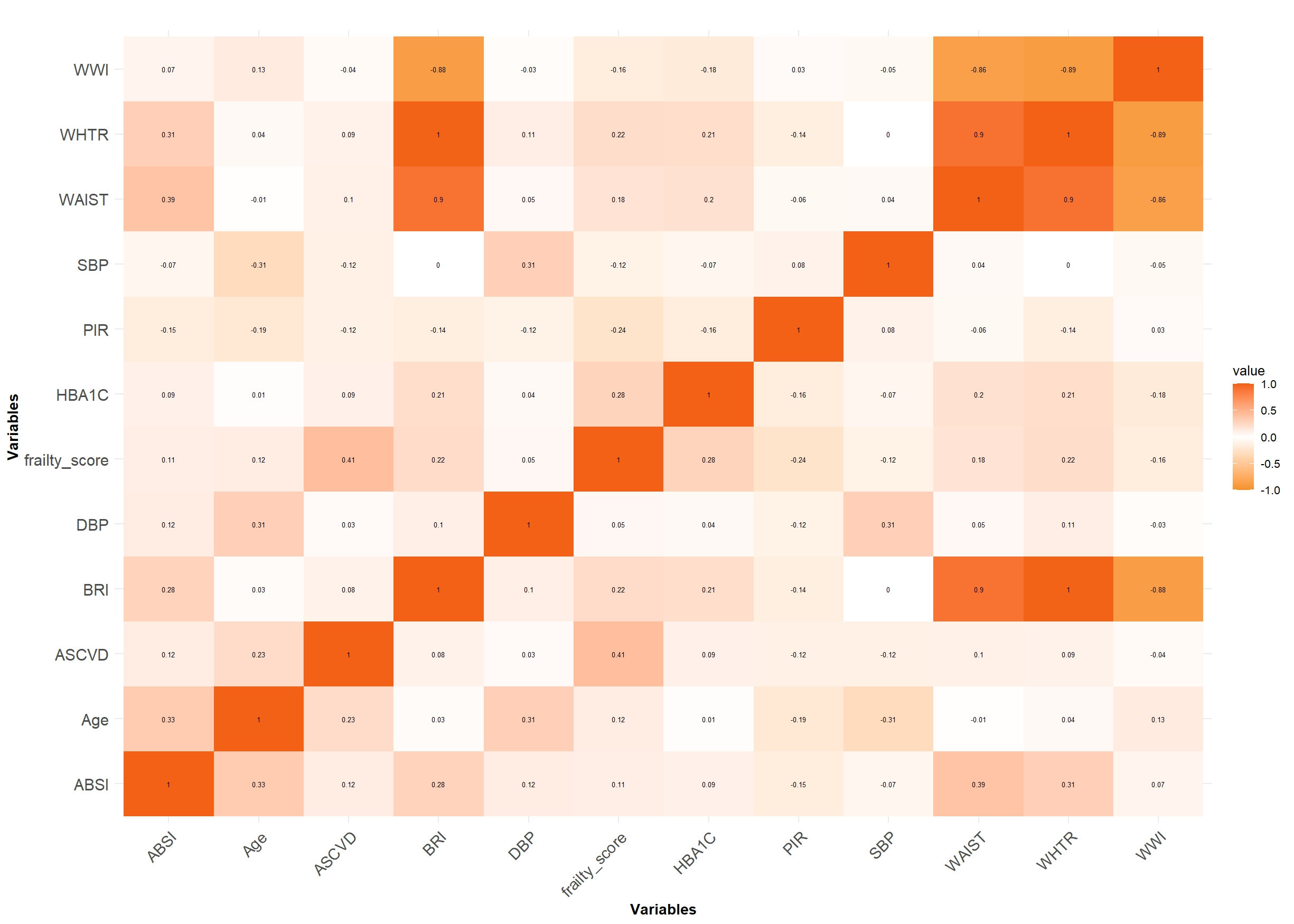

Supplement: Lin et al. supplementary material 2 — Lin et al. supplementary material [file S1463423626101364sup002.tiff]

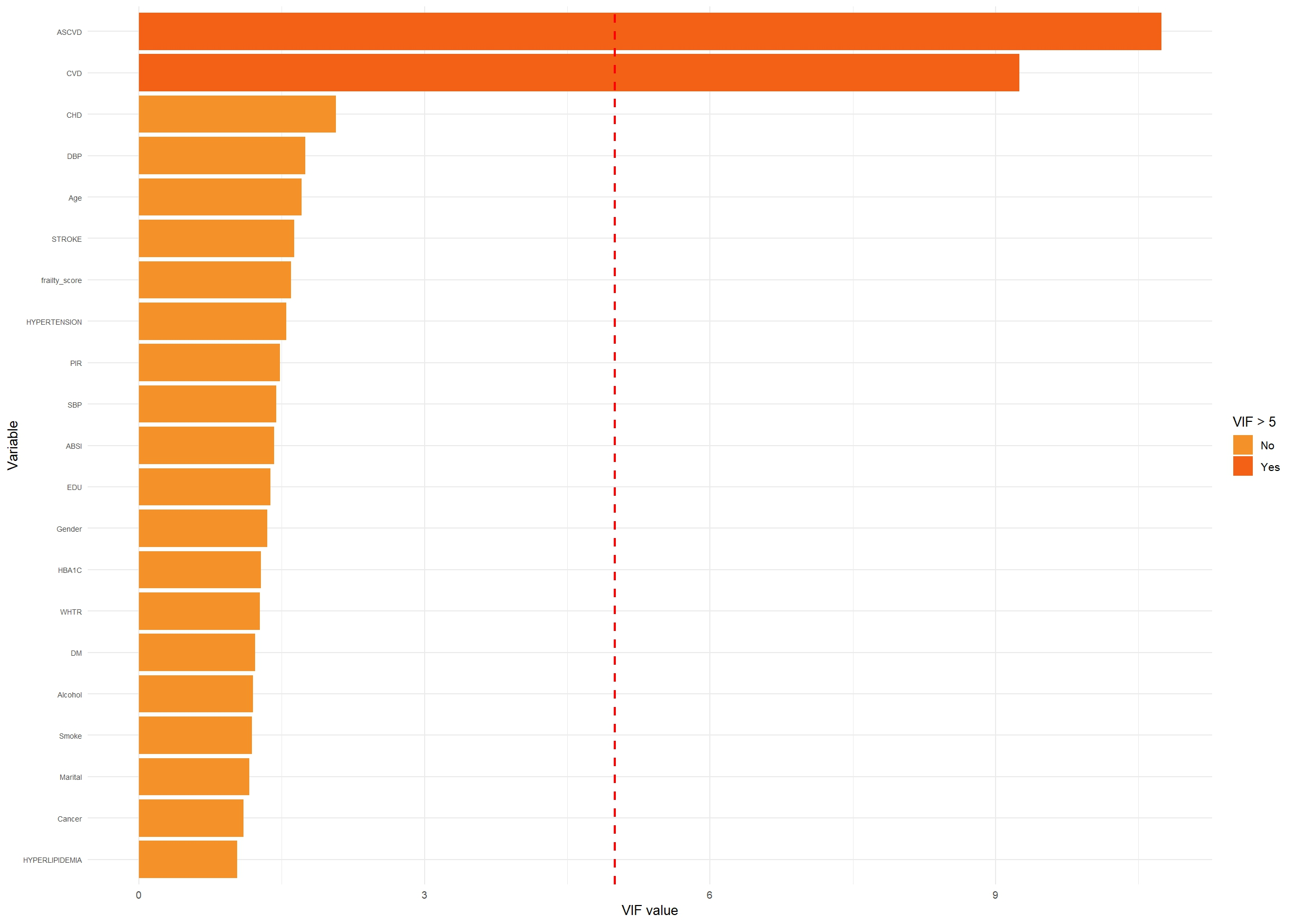

Supplement: Lin et al. supplementary material 3 — Lin et al. supplementary material [file S1463423626101364sup003.tiff]

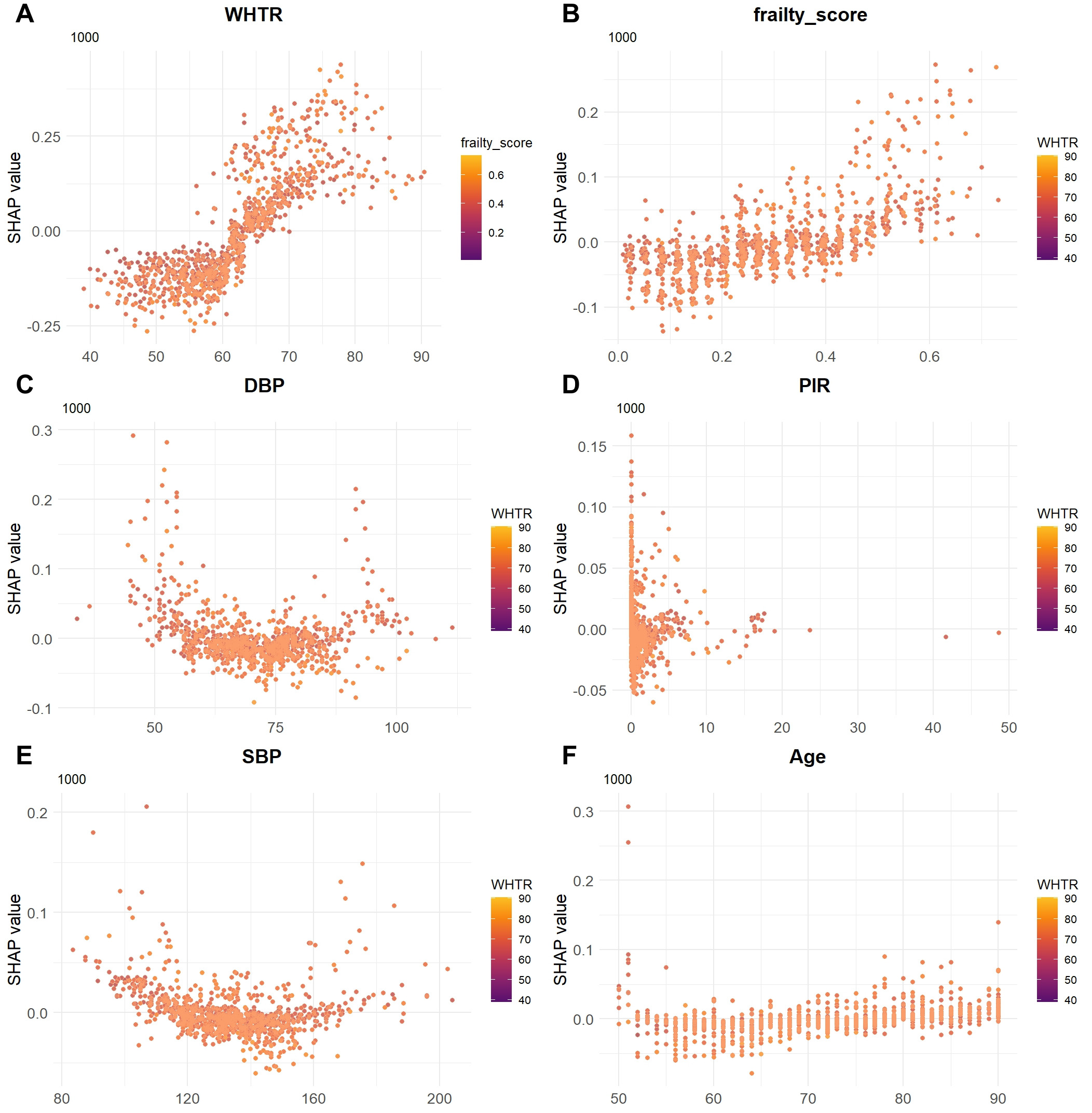

Supplement: Lin et al. supplementary material 4 — Lin et al. supplementary material [file S1463423626101364sup004.tiff]

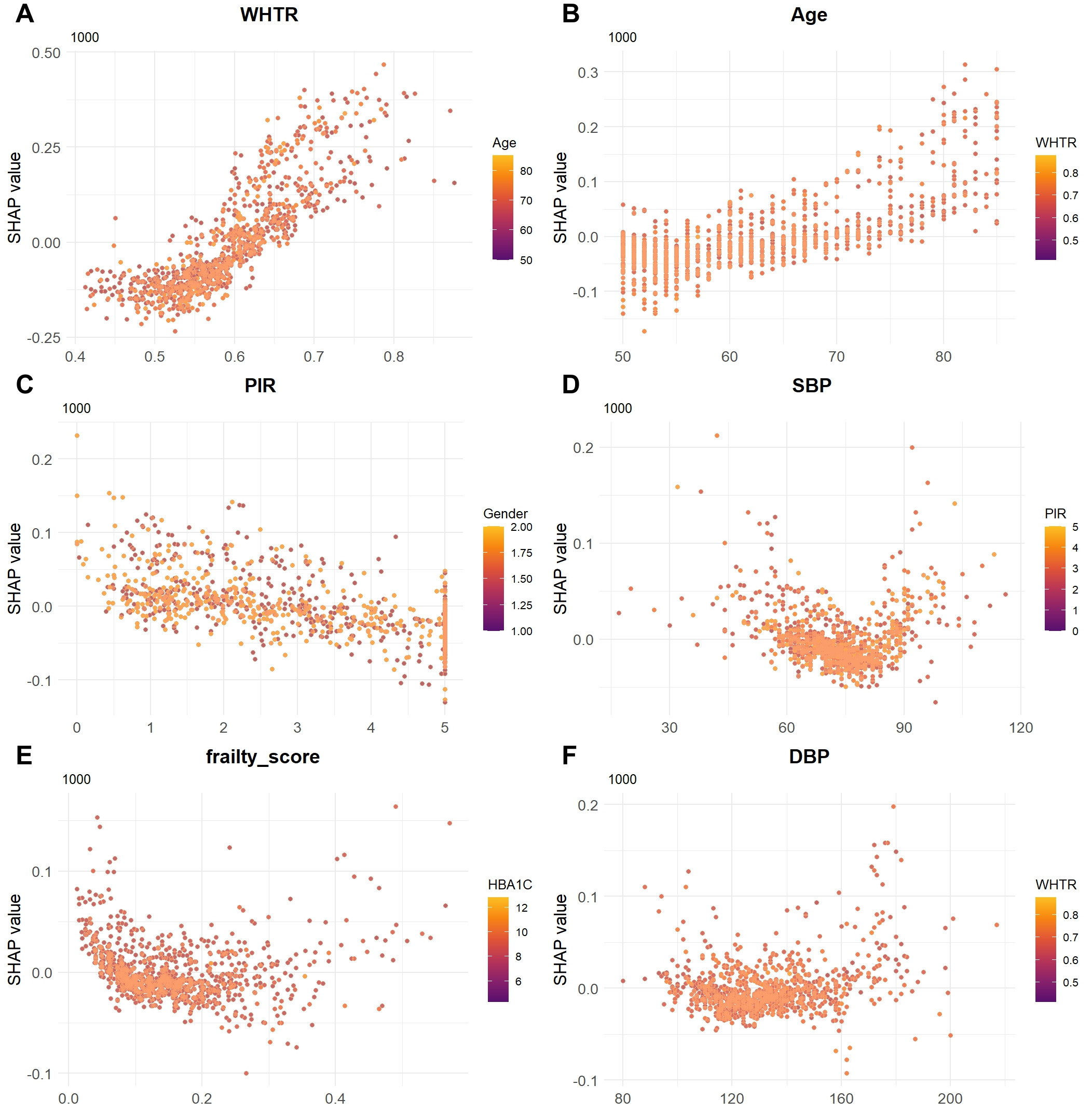

Supplement: Lin et al. supplementary material 5 — Lin et al. supplementary material [file S1463423626101364sup005.tiff]
