# Supplementary material for: Sarcopenia risk assessment among physically inactive middle-aged and older adults: interpretable machine-learning models in UK and US cohorts
Source: Prim Health Care Res Dev. 2026 Jun 24;27:e71. doi: 10.1017/S1463423626101364 (PMC13319488; doi:10.1017/S1463423626101364)
Supplement: Lin et al. supplementary material 6 — Lin et al. supplementary material [file S1463423626101364sup006.docx]

# **Supplementary Table 1:** Descriptive statistical analysis of relevant indicators of ELSA

| Variable | Overall | Non-sarcopenia | Sarcopenia | Statistic | P_Value |
| --- | --- | --- | --- | --- | --- |
| Gender | 1146 (100.00%) | 991 (86.5%) | 155 (13.5%) | 1.477 | 0.224 |
| Male | 416 (36.3%) | 367 (37.0%) | 49 (31.6%) |  |  |
| Female | 730 (63.7%) | 624 (63.0%) | 106 (68.4%) | |  |
| EDU | 1146 (100.00%) | 991 (86.5%) | 155 (13.5%) | 3.268 | 0.195 |
| Below high school | 654 (57.1%) | 560 (56.5%) | 94 (60.6%) |  |  |
| High school | 204 (17.8%) | 173 (17.5%) | 31 (20.0%) |  |  |
| Above high school | 288 (25.1%) | 258 (26.0%) | 30 (19.4%) |  |  |
| Marital | 1146 (100.00%) | 991 (86.5%) | 155 (13.5%) | 1.57 | 0.21 |
| No | 549 (47.9%) | 467 (47.1%) | 82 (52.9%) |  |  |
| Yes | 597 (52.1%) | 524 (52.9%) | 73 (47.1%) |  |  |
| STROKE | 1146 (100.00%) | 991 (86.5%) | 155 (13.5%) | 0.128 | 0.72 |
| No | 1026 (89.5%) | 889 (89.7%) | 137 (88.4%) | |  |
| Yes | 120 (10.5%) | 102 (10.3%) | 18 (11.6%) |  |  |
| DM | 1146 (100.00%) | 991 (86.5%) | 155 (13.5%) | 13.545 | <0.001 |
| No | 914 (79.8%) | 808 (81.5%) | 106 (68.4%) | |  |
| Yes | 232 (20.2%) | 183 (18.5%) | 49 (31.6%) |  |  |
| CHD | 1146 (100.00%) | 991 (86.5%) | 155 (13.5%) | 0.003 | 0.955 |
| No | 926 (80.8%) | 800 (80.7%) | 126 (81.3%) | |  |
| Yes | 220 (19.2%) | 191 (19.3%) | 29 (18.7%) |  |  |
| CVD | 1146 (100.00%) | 991 (86.5%) | 155 (13.5%) | 0 | 1 |
| No | 850 (74.2%) | 735 (74.2%) | 115 (74.2%) | |  |
| Yes | 296 (25.8%) | 256 (25.8%) | 40 (25.8%) |  |  |
| HYPERLIPIDEMIA | 1146 (100.00%) | 991 (86.5%) | 155 (13.5%) | 3.733 | 0.053 |
| No | 769 (67.1%) | 676 (68.2%) | 93 (60.0%) |  |  |
| Yes | 377 (32.9%) | 315 (31.8%) | 62 (40.0%) |  |  |
| Cancer | 1146 (100.00%) | 991 (86.5%) | 155 (13.5%) | 0.056 | 0.814 |
| No | 995 (86.8%) | 859 (86.7%) | 136 (87.7%) | |  |
| Yes | 151 (13.2%) | 132 (13.3%) | 19 (12.3%) |  |  |
| Alcohol | 1146 (100.00%) | 991 (86.5%) | 155 (13.5%) | 8.654 | 0.003 |
| No | 892 (77.8%) | 786 (79.3%) | 106 (68.4%) | |  |
| Yes | 254 (22.2%) | 205 (20.7%) | 49 (31.6%) |  |  |
| Smoke | 1146 (100.00%) | 991 (86.5%) | 155 (13.5%) | 0.668 | 0.414 |
| No | 327 (28.5%) | 278 (28.1%) | 49 (31.6%) |  |  |
| Yes | 819 (71.5%) | 713 (71.9%) | 106 (68.4%) | |  |
| HYPERTENSION | 1146 (100.00%) | 991 (86.5%) | 155 (13.5%) | 8.468 | 0.004 |
| No | 512 (44.7%) | 460 (46.4%) | 52 (33.5%) |  |  |
| Yes | 634 (55.3%) | 531 (53.6%) | 103 (66.5%) | |  |
| ASCVD | 1146 (100.00%) | 991 (86.5%) | 155 (13.5%) | 1.235 | 0.266 |
| No | 949 (82.8%) | 826 (83.4%) | 123 (79.4%) | |  |
| Yes | 197 (17.2%) | 165 (16.6%) | 32 (20.6%) |  |  |
| Age | 71.59 ± 10.60 | 71.55 ± 10.71 | 71.86 ± 9.92 | -0.363 | 0.717 |
| PIR | 1.33 ± 3.25 | 1.42 ± 3.44 | 0.75 ± 1.44 | 4.177 | <0.001 |
| Frailty score | 0.29 ± 0.16 | 0.28 ± 0.16 | 0.38 ± 0.15 | -7.441 | <0.001 |
| WHTR | 0.62 + 0.09 | 0.61 + 0.09 | 0.69 + 0.06 | -15.332 | <0.001 |
| WAIST | 100.44 ± 15.30 | 99.01 ± 15.24 | 109.56 ± 12.19 | -9.657 | <0.001 |
| BRI | 6.00 ± 2.12 | 5.72 ± 2.04 | 7.82 ± 1.67 | -14.05 | <0.001 |
| ABSI | 0.08 ± 0.01 | 0.08 ± 0.01 | 0.08 ± 0.01 | 2.525 | 0.012 |
| WWI | 23.46 ± 2.07 | 23.27 ± 2.08 | 24.68 ± 1.52 | -10.131 | <0.001 |
| SBP | 133.03 ± 18.54 | 133.09 ± 18.54 | 132.62 ± 18.65 | 0.29 | 0.772 |
| DBP | 70.97 ± 11.47 | 71.16 ± 11.39 | 69.77 ± 11.89 | 1.356 | 0.177 |
| HBA1C | 29.66 ± 23.56 | 29.59 ± 23.33 | 30.10 ± 25.03 | -0.237 | 0.813 |

Data are presented as n (%) for categorical variables and mean ± SD for continuous variables. Differences between participants with and without sarcopenia were assessed using the chi-square test for categorical variables and the independent t-test for continuous variables. Abbreviations: WHTR, waist-to-height ratio; BRI, body roundness index; ABSI, a body shape index; WWI, weight-adjusted waist index.
