# Supplementary material for: Sarcopenia risk assessment among physically inactive middle-aged and older adults: interpretable machine-learning models in UK and US cohorts
Source: Prim Health Care Res Dev. 2026 Jun 24;27:e71. doi: 10.1017/S1463423626101364 (PMC13319488; doi:10.1017/S1463423626101364)
Supplement: Lin et al. supplementary material 7 — Lin et al. supplementary material [file S1463423626101364sup007.docx]

# **Supplementary Table 2:** Descriptive statistical analysis of relevant indicators of NHANES

| Variable | Overall | Non-sarcopenia | Sarcopenia | Statistic | P_Value |
| --- | --- | --- | --- | --- | --- |
| Gender | 2733 (100.00%) | 2204 (80.6%) | 529 (19.4%) | 8.258 | 0.004 |
| Male | 1363 (49.9%) | 1069 (48.5%) | 294 (55.6%) | |  |
| Female | 1370 (50.1%) | 1135 (51.5%) | 235 (44.4%) | |  |
| Marital | 2733 (100.00%) | 2204 (80.6%) | 529 (19.4%) | 0.022 | 0.882 |
| No | 764 (28.0%) | 618 (28.0%) | 146 (27.6%) | |  |
| Yes | 1969 (72.0%) | 1586 (72.0%) | 383 (72.4%) | |  |
| EDU | 2733 (100.00%) | 2204 (80.6%) | 529 (19.4%) | 99.215 | <0.001 |
| Below high school | 409 (15.0%) | 266 (12.1%) | 143 (27.0%) | |  |
| High school | 980 (35.9%) | 771 (35.0%) | 209 (39.5%) | |  |
| Above high school | 1344 (49.2%) | 1167 (52.9%) | 177 (33.5%) | |  |
| Smoke | 2733 (100.00%) | 2204 (80.6%) | 529 (19.4%) | 0.186 | 0.666 |
| No | 1281 (46.9%) | 1038 (47.1%) | 243 (45.9%) | |  |
| Yes | 1452 (53.1%) | 1166 (52.9%) | 286 (54.1%) | |  |
| Alcohol | 2733 (100.00%) | 2204 (80.6%) | 529 (19.4%) | 5.665 | 0.017 |
| No | 375 (13.7%) | 285 (12.9%) | 90 (17.0%) |  |  |
| Yes | 2358 (86.3%) | 1919 (87.1%) | 439 (83.0%) | |  |
| HYPERTENSION | 2733 (100.00%) | 2204 (80.6%) | 529 (19.4%) | 13.287 | <0.001 |
| No | 1108 (40.5%) | 931 (42.2%) | 177 (33.5%) | |  |
| Yes | 1625 (59.5%) | 1273 (57.8%) | 352 (66.5%) | |  |
| DM | 2733 (100.00%) | 2204 (80.6%) | 529 (19.4%) | 24.052 | <0.001 |
| No | 2172 (79.5%) | 1793 (81.4%) | 379 (71.6%) | |  |
| Yes | 561 (20.5%) | 411 (18.6%) | 150 (28.4%) | |  |
| HYPERLIPIDEMIA | 2733 (100.00%) | 2204 (80.6%) | 529 (19.4%) | 2.367 | 0.124 |
| No | 538 (19.7%) | 447 (20.3%) | 91 (17.2%) |  |  |
| Yes | 2195 (80.3%) | 1757 (79.7%) | 438 (82.8%) | |  |
| CVD | 2733 (100.00%) | 2204 (80.6%) | 529 (19.4%) | 31.206 | <0.001 |
| No | 2319 (84.9%) | 1912 (86.8%) | 407 (76.9%) | |  |
| Yes | 414 (15.1%) | 292 (13.2%) | 122 (23.1%) | |  |
| CHD | 2733 (100.00%) | 2204 (80.6%) | 529 (19.4%) | 2.589 | 0.108 |
| No | 2565 (93.9%) | 2077 (94.2%) | 488 (92.2%) | |  |
| Yes | 168 (6.1%) | 127 (5.8%) | 41 (7.8%) |  |  |
| STROKE | 2733 (100.00%) | 2204 (80.6%) | 529 (19.4%) | 9.805 | 0.002 |
| No | 2621 (95.9%) | 2127 (96.5%) | 494 (93.4%) | |  |
| Yes | 112 (4.1%) | 77 (3.5%) | 35 (6.6%) |  |  |
| Cancer | 2733 (100.00%) | 2204 (80.6%) | 529 (19.4%) | 0.048 | 0.827 |
| No | 2366 (86.6%) | 1906 (86.5%) | 460 (87.0%) | |  |
| Yes | 367 (13.4%) | 298 (13.5%) | 69 (13.0%) |  |  |
| ASCVD | 2733 (100.00%) | 2204 (80.6%) | 529 (19.4%) | 27.327 | <0.001 |
| No | 2359 (86.3%) | 1940 (88.0%) | 419 (79.2%) | |  |
| Yes | 374 (13.7%) | 264 (12.0%) | 110 (20.8%) | |  |
| Age | 62.66 ± 9.93 | 61.70 ± 9.56 | 66.65 ± 10.42 | -9.972 | <0.001 |
| PIR | 3.00 ± 1.58 | 3.13 ± 1.58 | 2.44 ± 1.46 | 9.63 | <0.001 |
| WAIST | 99.22 ± 13.44 | 98.11 ± 13.31 | 103.82 ± 13.00 | -9.028 | <0.001 |
| WHTR | 0.60 ± 0.08 | 0.58 ± 0.08 | 0.65 ± 0.07 | -18.502 | <0.001 |
| WWI | 19.03 ± 1.74 | 19.19 ± 1.74 | 18.36 ± 1.57 | 10.779 | <0.001 |
| BRI | 5.45 ± 1.83 | 5.16 ± 1.71 | 6.68 ± 1.82 | -17.436 | <0.001 |
| ABSI | 0.08 ± 0.00 | 0.08 ± 0.00 | 0.08 ± 0.00 | -8.973 | <0.001 |
| SBP | 72.54 ± 12.22 | 72.91 ± 12.07 | 71.03 ± 12.70 | 3.082 | 0.002 |
| DBP | 132.38 ± 20.17 | 131.37 ± 20.03 | 136.56 ± 20.25 | -5.301 | <0.001 |
| HBA1C | 5.84 ± 1.08 | 5.80 ± 1.04 | 6.03 ± 1.20 | -4.089 | <0.001 |
| Frailty_score | 0.16 ± 0.09 | 0.15 ± 0.09 | 0.17 ± 0.10 | -4.401 | <0.001 |

Data are presented as n (%) for categorical variables and mean ± SD for continuous variables. Differences between participants with and without sarcopenia were assessed using the chi-square test for categorical variables and the independent t-test for continuous variables. Abbreviations: WHTR, waist-to-height ratio; BRI, body roundness index; ABSI, a body shape index; WWI, weight-adjusted waist index.
