# Supplementary material for: Sarcopenia risk assessment among physically inactive middle-aged and older adults: interpretable machine-learning models in UK and US cohorts
Source: Prim Health Care Res Dev. 2026 Jun 24;27:e71. doi: 10.1017/S1463423626101364 (PMC13319488; doi:10.1017/S1463423626101364)
Supplement: Lin et al. supplementary material 8 — Lin et al. supplementary material [file S1463423626101364sup008.docx]

**Supplementary Table 3: Performance of the ELSA Data Model on the Training Set (via Cross-Validation)**

| Model | Cross-validated AUC | Optimal hyperparameters |
| --- | --- | --- |
| Neural network | 0.8386 | hidden_units = 2.00; penalty = 0.077426; epochs = 560.00 |
| Random forest | 0.8367 | mtry = 7.00; trees = 1111.00; min_n = 2.00 |
| XGBoost | 0.8335 | trees = 2000.00; tree_depth = 11.00; learn_rate = 3.59e-03; loss_reduction = 4.64e-03 |
| Logistic regression | 0.8331 | penalty = 5.99e-03; mixture = 1.00 |
| Support vector machine (RBF) | 0.8282 | cost = 3.10e-03; rbf_sigma = 5.99e-03 |
| Decision tree | 0.7905 | cost_complexity = 1.00e-09; tree_depth = 13.00; min_n = 23.00 |
