# Supplementary material for: Sarcopenia risk assessment among physically inactive middle-aged and older adults: interpretable machine-learning models in UK and US cohorts
Source: Prim Health Care Res Dev. 2026 Jun 24;27:e71. doi: 10.1017/S1463423626101364 (PMC13319488; doi:10.1017/S1463423626101364)
Supplement: Lin et al. supplementary material 9 — Lin et al. supplementary material [file S1463423626101364sup009.docx]

**Supplementary Table 4: Performance of the NHANES Data Model on the Training Set (via Cross-Validation)**

| **Model** | **Cross-validated AUC** | **Optimal hyperparameters** |
| --- | --- | --- |
| Logistic regression | 0.7855 | penalty = 5.99e-03; mixture = 1.00 |
| Support vector machine (RBF) | 0.7803 | cost = 32.00; rbf_sigma = 4.64e-04 |
| XGBoost | 0.7765 | trees = 223.00; tree_depth = 1.00; learn_rate = 0.046416; loss_reduction = 1.29e-05 |
| Random forest | 0.7688 | mtry = 5.00; trees = 445.00; min_n = 35.00 |
| Neural network | 0.7661 | hidden_units = 4.00; penalty = 3.59e-05; epochs = 10.00 |
| Decision tree | 0.7390 | cost_complexity = 1.00e-08; tree_depth = 4.00; min_n = 35.00 |
